# Roles of Membrane and Vesicular Traffic in Regulation of the Hippo Pathway

**DOI:** 10.3389/fcell.2019.00384

**Published:** 2020-01-10

**Authors:** Shilpi Verghese, Ken Moberg

**Affiliations:** Department of Cell Biology, Emory University School of Medicine, Atlanta, GA, United States

**Keywords:** hippo signal pathway, vesicular trafficking, membrane localization, Yorkie, growth regulation

## Abstract

The Hippo pathway is a well conserved signaling cascade that modulates cell proliferation and survival in response to external cues such as cell:cell contact, injury, and nutritional status. Models of the Hippo pathway have evolved from a series of genetic interactions defined in the fruit fly *Drosophila melanogaster* into a complex series of biochemical mechanisms in which transmembrane and cytoskeletal proteins modulate cytoplasmic phosphatase and kinase activities that converge on the serine/threonine kinase Warts (Wts) to regulate nuclear entry of the co-activator protein Yorkie (Yki; vertebrate Yap1). This pathway is well conserved in human cells and broadly implicated in cancer. Progress in understanding biochemical events within the Hippo pathway highlights a need for improved understanding of the cell biological contexts in which these molecular interactions occur. A significant body of data linking Hippo signaling to membranes and proteins involved in intracellular membrane trafficking raise the possibility that some molecular regulatory events occur on the cytoplasmic face of vesicles. In *Drosophila*, a Yki-vesicle link was solidified by discoveries that cytoplasmic Yki concentrates at late-endosomes and physically interacts with two endosomal adaptor proteins, Myopic (Mop) and Leash. These two proteins are required for Yki to transit the endolysosomal pathway and be turned over in lysosomes. Molecules involved in recruiting and tethering Yki along this endosomal route are not defined but are predicted to play key roles in regulating Yki levels and thus Hippo-responsiveness of cells. As Wts is recruited to the apical membrane by upstream Hippo components, endosomal internalization could also affect complexes involved in Yki phosphorylation events that alter nucleocytoplasmic shuttling. Recent work has revealed an unexpected, non-transcriptional role of membrane-associated Yki in triggering actinomyosin contractility via the myosin-regulatory light chain Spaghetti squash (Sqh). How Yki interacts with the membrane and controls Sqh is unclear, but this mechanism represents a novel regulatory mechanism based on induced localization of Yki to a specific membrane compartment. These and other data will be discussed as we review data linking Yki to membrane and vesicular traffic in development and homeostasis and speculate on missing elements of these membrane-linked Yki regulatory mechanisms.

## Introduction

The Hippo pathway regulates organ size in diverse species from invertebrates to mammals (Pan, 2010; Zhao et al., 2010; Yu and Guan, 2013). The canonical pathway consists of a core kinase cascade comprising of Hippo/Mst1/2 (Hpo) (Harvey et al., 2003; Jia et al., 2003; Pantalacci et al., 2003; Udan et al., 2003) and Wts/Lats (Justice et al., 1995; Xu et al., 1995), along with their adaptor proteins Salvador/WW45 (Sav) (Kango-Singh et al., 2002; Tapon et al., 2002) and Mob kinase activator like 1/Mob1 (Lai et al., 2005). Wts/Lats phosphorylates the transcriptional co-activator Yorkie/YAP1 (Yes-associated protein-1) which recruits 14-3-3 proteins that in turn sequester Yki/YAP1 in the cytoplasm (Huang et al., 2005). Unphosphorylated Yki/YAP1 enters the nucleus and binds its cognate transcription factor (Scalloped/Sd in flies or TEADs in mammals) resulting in expression of target genes that regulate growth, proliferation, cell survival, metabolism, and stemness (Jukam et al., 2013). Several additional Hippo pathway components have been identified that regulate Yki activity once it has entered the nucleus, or by modulating Wts activity.

**FIGURE 1:**
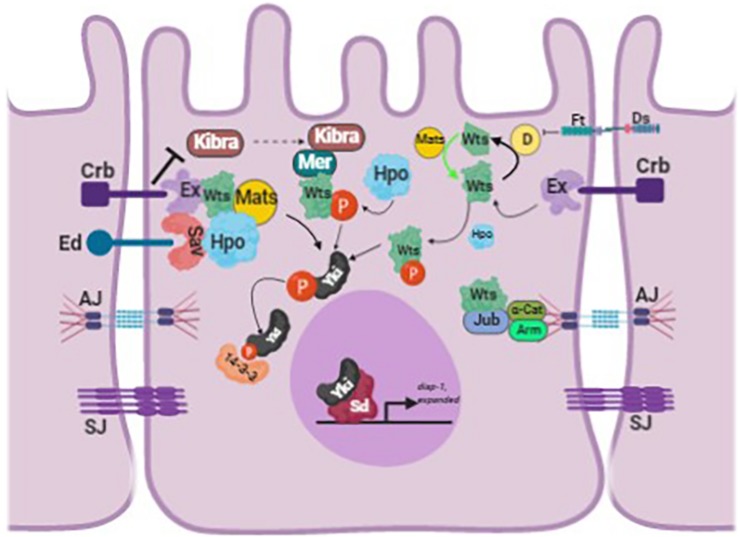
Schematic of the Hippo pathway in *Drosophila melanogaster*. The schematic shows how Yorkie is regulated by the different upstream regulators of the Hippo pathway. The figure is adapted and modified from Fulford et al. (2018). The different proteins abbreviated in the figure are: Crumbs (Crb), Echinoid (Ed), Expanded (Ex), Merlin (Mer), Warts (Wts), Salvador (Sav), Hippo (Hpo), Ajuba (Jub), Armadillo (Arm), α-Catenin (α-Cat), Yorkie (Yki), Scalloped (Sd), Adherens Junction (AJ), and Sepatate Junction (SJ) (image created with BioRender).

Because upstream Hippo signals converge on a single process, the nucleocytoplasmic shuttling of Yki/Yap1, the cytoplasmic Yki/Yap1 pool is generally regarded an inactive reservoir of transcriptional potential that can be mobilized into the nucleus by various triggers (Figure 1). This regulatory step is sufficiently generalized such that the relative ratio of cytoplasmic:nuclear Yki/Yap1 is used as a convenient readout of upstream Hippo activity in various experimental systems. However, this perspective needs to be expanded somewhat to appreciate a body of data that indicate Yki/Yap1 is not diffusely localized in the cytoplasm but is rather highly enriched at specific membranous structures within cells, including vesicles of the endolysosomal system. Moreover, experimental evidence indicates that this colocalization with endosomes is required for Yki/Yap1 protein turnover and may also contribute to Yki/Yap1 regulation by Wart/Lats. Thus, cytoplasmic Yki/Yap1 is not a free-floating, inert pool of protein but is tethered to specific intracellular membranes, and dynamically trafficked through vesicular compartments that may represent sites at which Yki/Yap1 is exposed to inhibitory signals from the Hippo pathway. Factors that control Yki/Yap1 entry into and traffic through the endolysosomal system may thus modify Yki/Yap1 activity but are not well understood. Below we review data on the relationship between vesicle-associated factors and Yki/Yap1, the apparent roles of these factors in modulating Yki/Yap1 activity and speculate on the broader impacts of vesicle trafficking on Hippo pathway components and activity.

## General Endocytic Proteins in Yorkie Regulation

The set of endosomal factors that modulate Yki activity in wing epithelial cells includes proteins that play general roles in internalizing and routing membrane-associated complexes into endosomes. These general factors include the adaptor complex subunit AP-2σ, the syntaxin Syx7/Avalanche (Avl), the early endosomal GTPase Rab5, and the Vps23/Erupted and Vps25 ESCRT (endosomal sorting complex required for transport) components (Lu and Bilder, 2005; Robinson and Moberg, 2011). AP-2σ is a subunit of the AP-2 complex, which recruits transmembrane proteins into clathrin-coated pits during early stages of endocytic internalization from the plasma membrane (PM) (reviewed in Schmid and McMahon, 2007). *Syx7/avalanche* encodes a SNARE protein that functions with Rab5 to promote fusion of newly internalized endocytic vesicles to early endosome (Lu and Bilder, 2005). The ESCRT factors Erupted/Vps23 and Vps25 are required to form perinuclear multivesicular bodies [MVBs; aka late endosomes (LEs)] by inward budding of endosomal membranes prior to fusion with lysosomes (Saksena et al., 2007) and also act at the midbody to control abscission after cytokinesis (Gulluni et al., 2017). RNAi knockdown of any of these general endosomal trafficking factors activates the Yki reporter *expanded-lacZ* (*ex-lacZ*) in larval wing cells. Thus, targeted loss of several endolysosomal factors elevates nuclear Yki activity, and this link is detected at multiple steps along the endolysosomal route, including cell-surface internalization, early endosome formation and perinuclear MVB biogenesis. One interpretation of these data is that internalization of an Yki-regulatory complex at the PM, perhaps in association with a transmembrane Hippo component, and subsequent trafficking of this complex through MVBs is required to limit Yki nuclear activity among cells in an intact epithelial sheet.

## Yki Physical Links to Endosomal Membranes

The effect of general endocytic factors like *erupted* and *avalanche* on Yki activity suggested that Yki itself might be trafficked into endolysosomal compartments, perhaps in complex with the transmembrane Hippo proteins Echinoid (Ed) and Crumbs (Crb), which respectively interact with Salvador (Sav) and Expanded (Ex), and regulate Yki activity (Robinson et al., 2010; Yue et al., 2012). The first element of this hypothesis was confirmed by a detailed analysis of Yki subcellular localization in *Drosophila* wing cells that found Yki concentrates in discrete puncta that colocalize with the LE marker Rab7 (Gilbert et al., 2011). However, subsequent studies found that this Yki-endosome link was found to depend in part on two previously unidentified Yki regulators, Mopand Leash, which are endosome-resident proteins that interact with Yki and recruit it to specific vesicular membranes.

### Myopic

Myopic (Mop) is a *Drosophila* homolog of the tumor suppressor His-domain protein tyrosine phosphatase (HD-PTP; Kok et al., 1997; Szeles et al., 1997; Toyooka et al., 2000; Braga et al., 2002; Manteghi et al., 2016) and contains an amino terminal Bro-1 domain that interacts with endosomal proteins, e.g., CHMP4b (Odorizzi et al., 2003; Kim et al., 2005; Ichioka et al., 2007; Doyotte et al., 2008; Miura et al., 2008), and a carboxy domain with sequence homology to protein tyrosine phosphatases. Mop had previously been shown to regulate EFGR trafficking (Miura et al., 2008) but Gilbert et al. found that the intervening linker region of Mop contains two PPxY (proline-proline-X-tyrosine) motifs that allow Mop to bind to Yki. In addition to these PPxY motifs, the Mop linker region also contains a predicted Alix V domain. This domain was originally found in the endosomal protein Alix and is composed of three helical bundles that adopt a “V” shape that interact with HIV Gag proteins involved in viral budding (Fisher et al., 2007). The Mop PPxY motifs are located just downstream of this Alix V domain. Complementary co-localization data suggest that a Mop-Yki complex exists on the outer surface of Rab7-positive late endosomes. Disrupting this complex by removing Mop shifts Yki onto Rab5-positive early endosomes while at the same time elevating Yki levels and nuclear activity. Thus, Mop appears to be a key physical link between Yki and the LE compartment. Notably, Mop can inhibit Yki activity independent of Warts, which is consistent with physical binding of Mop and Yki.

### Leash

The *Drosophila* Leash protein (CG4674) is a member of the vesicle-associated arrestin domain containing (Arrdc) protein family and was identified as a Yki-binding protein in an interaction screen (Kwon et al., 2013). Leash co-precipitates with Yki, and the two proteins colocalize on Rab9-positive LEs. Overexpression of Leash, or it’s human homolog Arrdc3, suppressed Yki-driven proliferation of intestinal stem cells, while loss of Leash elevates Yki protein levels and activity and at the same time reducing Yki vesicular association, implicating Leash in control of Yki endolysosomal traffic. Yki-containing puncta are sometimes encircled by the lysosomal marker Lamp1, indicating that the lysosome is indeed one terminal destination of endosome-associated Yki. In support of this model, bafilomycin, an inhibitor of lysosomal acidification, increased Yki activity and reversed the effect of Leash over expression. A recent study has confirmed the corresponding Arrdc3-Yap1 interaction in human cells (Shen et al., 2018). Intriguingly, the authors of the Leash study also found that overexpression of Wts could increase the abundance of Yki-positive vesicles in cultured cells, prompting the hypothesis that Yki endolysosomal trafficking may be *regulated* by the canonical Hippo pathway, rather than operating in parallel to it. If confirmed, this result would imply that Hippo signals not only regulate Yki phosphorylation and nucleocytoplasmic shuttling, but also Yki traffic along an endosomal route that could deliver Yki to perinuclear LE/MVB compartments, perhaps poising Yki for either of two opposing fates: lysosomal turnover or nuclear entry. Notably, the same screen that identified Leash also identified and validated the Sec31 protein as an Yki interacting protein. Sec 31 encodes and essential component of the COPII coat required for transport vesicle budding from the endoplasmic reticulum (Salama et al., 1997), which could indicate Yki interacts with proteins in a number of different intracellular membrane trafficking systems with as yet undetermined consequences.

## Vertebrate Yap1 Is Regulated by the Early Endosomal Protein Endotubin (GP150)

Similar to *Drosophila* Yki, vertebrate Yap1 has also been found to be regulated by resident endosomal proteins. One of these factors is Endotubin [Edtb; aka Apical Early Endosomal Glycoprotein (AEEG)], a glycosylated integral-membrane protein concentrated in tubular endosomes in the apical cortex of polarized epithelial cells (Wilson et al., 1987; Trahair et al., 1995; McCarter et al., 2010). Yap1 and Edtb colocalize on apical endosomes in Madin-Darby canine kidney (MDCK) cells and Edtb competes with Yap1 for binding to Angiomotin (Amot) (Cox et al., 2015). Amot was initially identified due to an interaction with angiostatin and has since been shown to be enriched at cell junctions, where it interacts with the actin cytoskeleton, the Rho regulator Cdc42 RhoGAP, and the Patj-Pals polarity complex to coordinate epithelial cell polarity (Chan et al., 2011). The interaction with Amot thus sequesters Yap1 in the cytoplasm. Over-expression of Edtb displaces Yap1 from Amot1 and results in enhanced Yap1 nuclear localization, elevated cell proliferation, and clonal outgrowth in soft agar. Intriguingly, the relative distribution of Etdb:Amot ⇌ Amot:Yap1 complexes is influenced by cell confluency – Edtb preferentially associates with Amot proteins at the early endosome compartment of subconfluent cells and promotes Yap1 nuclear localization and proliferation. Importantly, this study also found that shRNA to Edtb decreases nuclear Yap1, indicating that endogenous Edtb normally promotes Yap1 nuclear translocation, perhaps via contact inhibition signals transduced through the canonical Hippo pathway.

## Yki/Yap1 Regulation by Phospholipids

Each cellular membrane is composed of a distinct mixture of phosphoinositide (PI) lipids that impart specific functions and properties to the corresponding membrane. One group of enzymes responsible for PI synthesis are lipid kinases, which attach phosphate groups to phosphatidylinositol (PI) at positions 3, 4, or 5 of the inositol ring. One of these lipid kinases, PI4KIIIa, catalyzes the synthesis of phosphatidylinositol-4-phosphate, PI(4)P, which is a component of some membranes, including the PM, and a precursor of more complex phosphoinositides. Mutations in the *Drosophila PI4KIII*α gene (CG10260) were identified in a genetic screen for factors required in follicle epithelial cells for *Drosophila* oocyte polarization (Yan et al., 2011). These *PI4KII*α oocyte polarization defects phenocopy mutations in Hippo components, which led the authors to examine the effects of *PI4KIII*α alleles on Hippo signaling. Remarkably, they found that *PI4KIII*αloss prevents the Hippo component Merlin (Mer) (Neurofibromatosis Type-2 or NF2 in humans) from localizing to the apical domain, which consequently impairs Hippo signaling and activates Yki. These data indicate that Mer retention at the apical PM requires PI(4)P or its derivatives, and raises the issue of whether other Hippo components also interact with phospholipids. More recently, a proximity biotinylation screen for proteins that associate with phosphatidylserine (ptd-Ser), which is enriched in the cytoplasmic leaflet of the recycling endosomes (REs), identified Yap1 as a major “hit” (Matsudaira et al., 2017). Yap1 was found to localize to REs in sub-confluent in Cos and HEK293A cells, but disperse from REs when cells reach confluency, again suggesting that contact inhibition signals transduced through the Hippo pathway alter Yap1 trafficking though the endosomal pathway. Remarkably, a comparison of the localization of total Yap1 and pSer127-Yap1 (i.e., phosphorylated on Serine 127 by Lats) in sub-confluent cells indicated that RE-associated Yap1 is mainly unphosphorylated, and thus may be en route to the nucleus.

To directly test the role of ptd-Ser in Yap1 localization and activity, the authors manipulated ATP8A1, a RE-enriched flippase that catalyzes the enrichment of ptd-Ser on the outer leaflet of the RE membrane (Roux et al., 2012). Remarkably, siRNAs to *ATP8A1* increased pSer217-Yap1 and reduced nuclear localization and activity of Yap1. Importantly, manipulating ptd-Ser levels by three other approaches – masking ptd-Ser with a PH domain, metabolic depletion of ptd-Ser, or expression of a ptd-Ser degrading enzyme – all produced similar effects on Yap1 in cultured cells. Thus, the link between ptd-Ser and Yap1/Hippo is robust in these experimental systems. A related series of experiments in this same study identified another RE-resident protein named Evectin-2 as a regulator of Yap1 activity. However, additional work found that Evectin-2 regulates ubiquitin-mediated degradation of Lats1 via a mechanism that is independent of the effects of ATP8A1/PS on Yap1. In sum, these data provide two potential mechanisms through which proteins that localize to REs regulate Yap1 and Hippo activity in mammalian cells. It would be significant to identify contexts in which either or both of these RE-based regulatory mechanisms modulate Yap1/Hippo activity in development or disease.

## Regulation of Yap1 by the Le-Associated Protein Lamtor1/p18

A recent study (Block et al., 2019) in cultured vertebrate cells provides preliminary evidence of an additional link between Yap1 and late endosomes through a protein termed p18/LAMTOR1 (CG14184 in *Drosophila*) which is proposed to regulate a “long recycling loop” involving detergent resistant membranes (DRMs). In human and mouse cells, p18/LAMTOR1 serves as platform for TORC1 signaling and other pathways that function via late endosomes/lysosomes (Mu et al., 2017). Loss of p18/LAMTOR1 in mesenchymal osteoblast cells shifts Yap1 out of the nucleus and results in Yap1 hyperphosphorylation, as does primaquine, an inhibitor of the late RE loop. These effects of p18/LAMTOR1 on Yap1 are proposed to occur via altered trafficking of the Src kinase, which is implicated in Yap1 regulation by integrin complexes. β1-integrin and p18/LAMTOR1 stimulate Src recycling to the PM, which in turn enhances Yap1 nuclear localization. These data suggest that a p18/LAMTOR endosome recycling mechanism plays a key role in controlling Yap1 downstream of integrin-mediated adhesion of cultured cells, but this model will need be confirmed and validated with *in vivo* systems.

## Non-Transcriptional Role of Yorkie/YAP at Membranes

Although Yki/Yap1 were originally identified for their roles as nuclear transcriptional co-activators, an emerging body of work suggests that the “inactive” cytoplasmic pools of these proteins actually have important, non-transcriptional roles at membranes. For example, a fraction of cytoplasmic Yap1 localizes to the membrane constriction at the midbody during cytokinesis of human cell lines (e.g., MCF-10A and Hela) and is required for abscission via a transcription-independent mechanism (Bui et al., 2016). In these cells, siRNA knockdown of Yap mislocalizes proteins that regulate actin-based contraction during cytokinesis (e.g., RhoA) and increased the phosphorylation of myosin light chain (MLC), which activates the contractile motor myosin-II. A more recent study in *Drosophila* has found evidence of a similar link between membrane-associated Yki and myosin-contractility in polarized wing epithelial cells (Xu et al., 2018). In this study, the authors found that Hippo inactivation enhances localization of Yki to apical junctions, and that direct tethering Yki to the apical membrane with a myristoyl tag (myr-Yki) leads to tissue deformation, suggestive of elevated cortical tension. This effect on epithelial architecture correlated with phosphorylation and activation of the myosin-II regulatory light chain (MRLC) factor Spaghetti Squash (Sqh) via the MRLC kinase Stretchin-Mlck in a Sd-independent, and thus likely transcription-independent, manner. This in turn increased actinomyosin contractility, which is proposed to act in a feed-forward loop to enhance nuclear Yap1 activity.

In summary, these Yap1 and Yki data provide evidence of an underappreciated and perhaps generalized role for cytoplasmic Yki/Yap1 in stimulating cortical tension and contractility. But key questions remain. For example, what factors normally tether Yki to apical junctions in cells with reduced Hippo signals? Is this role filled by Ex, or by a membrane-associated factor akin to Mop? And how does myr-Yki promote the activity of kinases that phosphorylate Sqh? Future studies will help answer these questions and, inevitably, raise others.

## Summary

**FIGURE 2:**
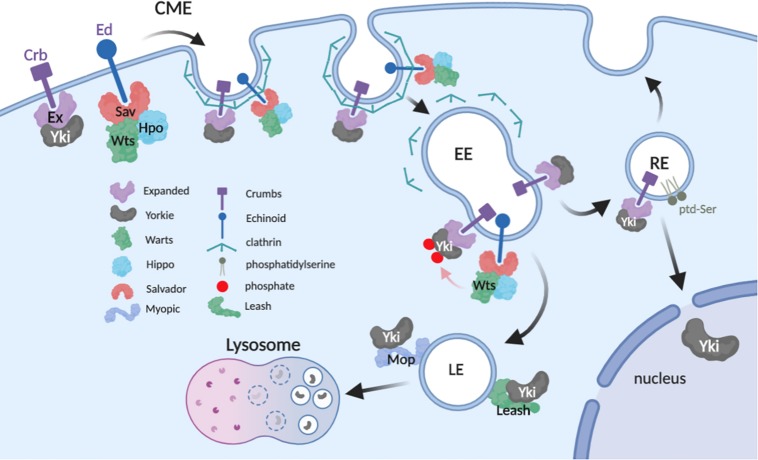
Proposed model of Yki regulation in the endolysosomal system. The model incorporates potential interactions between Yki and regulators while in transit from apical plasma membrane (PM) to distal vesicular compartments (image created with BioRender).

The data reviewed here show a consistent pattern of physical and functional interactions between Yki/Yap1 and proteins distributed throughout the endolysosomal system. One consistent theme of these studies is that internalized Yki/Yap1 are ultimately routed into lysosomes and degraded. This mechanism in part explains endocytic factors that inhibit Yki/Yap1 (e.g., Mop and Leash), but it does not explain those that promote Yki/Yap1 nuclear localization (e.g., Edtb and Atp8A1). These divergent effects could simply reflect cell-type differences in how Yki/Yap1 are regulated by endocytosis. However, an alternative explanation may be that endocytosis can affect Yki/Yap1 through multiple mechanisms in a single cell, perhaps even simultaneously (Figure 2). One of these mechanisms is clearly Yki/Yap1 degradation in the terminal endolysosomal compartment, the lysosome. Another may be that endocytosis is necessary to fully expose Yki/Yap1 to inhibitory Hippo signals in distal endosomal compartments, which could explain the observed effects of some endocytic factors on Wts/Lats phosphorylation of Yki/Yap1. Finally, a significant body of genetic and molecular data support a role for endosomes as key signaling centers for signal transduction pathways that influence the nuclear translocation of latent cytoplasmic transcription factors (e.g., see Miaczynska et al., 2004; Murphy et al., 2009; Taelman et al., 2010). It thus seems reasonable to consider the hypothesis that endocytosis is necessary to traffic Yki/Yap1 into perinuclear vesicular compartments that poise Yki/Yap1 for nucleocytoplasmic shuttling. In support of this model, microtubule-regulated transport of Mer to the nuclear periphery (Miaczynska et al., 2004; Murphy et al., 2009; Taelman et al., 2010) controls Yki nucleocytoplasmic shuttling of Yki (Bensenor et al., 2010). As Mer modulates internalization of transmembrane receptors (for review see McClatchey and Fehon, 2009), this mechanism could control endosomal internalization and transit of Yki:receptor complexes that eventually release Yki to enter the nucleus. Molecular mechanisms that release Yki from endosomes are not known – however, the effect of the RE lipid ptd-Ser on Yap1 phosphorylation and nuclear entry implies that an as yet unidentified ptd-Ser-binding protein has a role in this process.

The direct Yki-interactors Mop and Leash seem logical candidates to recruit and retain Yki on endosomes as it transits the endolysosomal system. However, analysis of Mop and Leash mutant cells reveal that neither protein is actually required to sequester Yki on a membrane; loss of either factor does not disperse Yki from membranes but simply shifts it to a different vesicular compartment. Thus, other factor(s) must serve a Yki membrane-tether role. One candidate Yki membrane-tether is the Hippo receptor Ed, which interacts with Sav, Mer, Kibra, and Yki to promote Yorkie phosphorylation, and has been speculated to recruit associated factors into endosomes for recycling or lysosomal turnover (Yue et al., 2012). However, a number of interactions between Yki and Hippo components (e.g., Wts) depend on the same PPxY:WW domain interaction module as that used by Myopic, implying a handover of Yki to in a post-PM compartment. Thus, it seems likely that as yet unidentified factors may be required to retain Yki/Yap1 on PS-containing endosomal membranes.

Finally, it is interesting to consider what mechanisms may act as apical triggers of Yki internalization from the PM. In *Drosophila* wing epithelial cells, Yki is recruited to the adherens junctions by interaction with the FERM-domain protein Expanded (Badouel et al., 2009) which is proposed to bring Yki into proximity to Warts-containing regulatory complexes anchored to the membrane by transmembrane proteins such as Ed (Yue et al., 2012). Since Wts overexpression enhances Yki localization to endosomes in larval wing cells (Kwon et al., 2013), Yki internalization on endosomes may simply be a consequence of phosphorylation by Wts. This kind of mechanism could explain why cytoplasmic Yki concentrates at apical junctions, rather than endosomes, in *wts* deficient cells (Xu et al., 2018). However, it also seems likely that the continual endocytosis of apical membrane results in a steady flow of Yki:receptor complexes entering EEs, which either return to the PM via REs, or enter LEs and the lysosome. Finally, the regulated internalization and degradation of some receptors, e.g., Notch internalization driven by mono-ubiquitination by the Itch/NEDD4/Su(dx) family of HECT domain E3 ligases (reviewed in Bray, 2006) raises the possibility that a similar mechanism may serve as a molecular trigger that controls abundance of Yki:receptor complexes on the PM.

## Conclusion

In conclusion, a review of the literature provides strong evidence that endosomal trafficking plays a role in regulating the Hippo pathway generally, and specifically in controlling the localization, levels and activity of the Yki/Yap1 proteins. Molecules involved in directing Yki onto budding endosomes and tethering it along the endosomal route are not well defined but are predicted to play key roles in regulating Yki levels and thus Hippo-responsiveness of cells (Table 1). A better understanding of this process and how it is regulated could yield new insights into how Hippo activity is modulated in developing tissues and provide avenues for therapeutic strategies that aim to reduce Yap1 activity and levels in human cancers.

**TABLE 1:** Selected Yki/Yap1 interactors and regulators that locate to endosomes.

Interactor	Compartment	Type of interaction	Interation domain/motif	Organism	References
Myopic	Rab7 late endosome	Physical complex	PPPY^791^ and PPAY^823^	*Drosophila*	Gilbert et al., 2011
Leash/ARRDC3	Rab9 late endosome	Physical complex	Arrestin domains aa 1–305	*Drosophila* and human	Kwon et al., 2013
ptds-Serine	Recycling endosomes	Likely indirect	Not determined	Monkey and human	Matsudaira et al., 2017
p18/LAMTOR	Late endosomes	Indirect	Not determined	Mouse	Block et al., 2019

*Different proteins either directly or indirectly interact with Yki/YAP to localize it on the endosomes from endosomal trafficking.*

## Author Contributions

SV and KM wrote and edited the review.

## Conflict of Interest

The authors declare that the research was conducted in the absence of any commercial or financial relationships that could be construed as a potential conflict of interest.

## References

[B1] Badouel C. Gardano L. Amin N. Garg A. Rosenfeld R. Le Bihan T. (2009). The FERM-domain protein expanded regulates Hippo pathway activity via direct interactions with the transcriptional activator Yorkie. *Dev. Cell* 16 411–420. 10.1016/j.devcel.2009.01.010 19289086

[B2] Bensenor L. B. Barlan K. Rice S. E. Fehon R. G. Gelfand V. I. (2010). Microtubule-mediated transport of the tumor-suppressor protein merlin and its mutants. *Proc. Natl. Acad. Sci. U.S.A.* 107 7311–7316. 10.1073/pnas.0907389107 20368450 PMC2867680

[B3] Block M. R. Brunner M. Ziegelmeyer T. Lallemand D. Pezet M. Chevalier G. (2019). Integrin-dependent YAP signaling requires LAMTOR1 mediated delivery of Src to the plasma membrane. *bioRxiv* [Preprint]. 10.1101/585349.

[B4] Braga E. Senchenko V. Bazov I. Loginov W. Liu J. Ermilova V. (2002). Critical tumor-suppressor gene regions on chromosome 3P in major human epithelial malignancies: allelotyping and quantitative real-time PCR. *Int. J. Cancer* 100 534–541. 10.1002/ijc.10511 12124802

[B5] Bray S. J. (2006). Notch signalling: a simple pathway becomes complex. *Nat. Rev. Mol. Cell Biol.* 7 678–689. 10.1038/nrm2009 16921404

[B6] Bui D. A. Lee W. White A. E. Harper J. W. Schackmann R. C. Overholtzer M. (2016). Cytokinesis involves a nontranscriptional function of the Hippo pathway effector YAP. *Sci. Signal.* 9:ra23. 10.1126/scisignal.aaa9227 26933062 PMC5455055

[B7] Chan S. W. Lim C. J. Chong Y. F. Pobbati A. V. Huang C. Hong W. (2011). Hippo pathway-independent restriction of TAZ and YAP by angiomotin. *J. Biol. Chem.* 286 7018–7026. 10.1074/jbc.C110.212621 21224387 PMC3044958

[B8] Cox C. M. Mandell E. K. Stewart L. Lu R. Johnson D. L. McCarter S. D. (2015). Endosomal regulation of contact inhibition through the AMOT:YAP pathway. *Mol. Biol. Cell* 26 2673–2684. 10.1091/mbc.E15-04-0224 25995376 PMC4501364

[B9] Doyotte A. Mironov A. McKenzie E. Woodman P. (2008). The Bro1-related protein HD-PTP/PTPN23 is required for endosomal cargo sorting and multivesicular body morphogenesis. *Proc. Natl. Acad. Sci. U.S.A.* 105 6308–6313. 10.1073/pnas.0707601105 18434552 PMC2359801

[B10] Fisher R. D. Chung H. Y. Zhai Q. Robinson H. Sundquist W. I. Hill C. P. (2007). Structural and biochemical studies of ALIX/AIP1 and its role in retrovirus budding. *Cell* 128 841–852. 10.1016/j.cell.2007.01.035 17350572

[B11] Fulford A. Tapon N. Ribeiro P. S. (2018). Upstairs, downstairs: spatial regulation of Hippo signalling. *Curr. Opin. Cell Biol.* 51 22–32. 10.1016/j.ceb.2017.10.006 29154163

[B12] Gilbert M. M. Tipping M. Veraksa A. Moberg K. H. (2011). A screen for conditional growth suppressor genes identifies the drosophila homolog of HD-PTP as a regulator of the oncoprotein Yorkie. *Dev. Cell* 20 700–712. 10.1016/j.devcel.2011.04.012 21571226 PMC3386645

[B13] Gulluni F. Martini M. Hirsch E. (2017). Cytokinetic abscission: phosphoinositides and ESCRTs Direct the final cut. *J. Cell Biochem.* 118 3561–3568. 10.1002/jcb.26066 28419521

[B14] Harvey K. F. Pfleger C. M. Hariharan I. K. (2003). The drosophila mst ortholog, Hippo, restricts growth and cell proliferation and promotes apoptosis. *Cell* 114 457–467. 10.1016/s0092-8674(03)00557-9 12941274

[B15] Huang J. Wu S. Barrera J. Matthews K. Pan D. (2005). The Hippo signaling pathway coordinately regulates cell proliferation and apoptosis by inactivating Yorkie, the drosophila homolog of YaP. *Cell* 122 421–434. 10.1016/j.cell.2005.06.007 16096061

[B16] Ichioka F. Takaya E. Suzuki H. Kajigaya S. Buchman V. L. Shibata H. (2007). HD-PTP and Alix share some membrane-traffic related proteins that interact with their bro1 domains or proline-rich regions. *Arch. Biochem. Biophys.* 457 142–149. 10.1016/j.abb.2006.11.008 17174262

[B17] Jia J. Zhang W. Wang B. Trinko R. Jiang J. (2003). The drosophila Ste20 family kinase dMST functions as a tumor suppressor by restricting cell proliferation and promoting apoptosis. *Genes. Dev.* 17 2514–2519. 10.1101/gad.1134003 14561774 PMC218145

[B18] Jukam D. Xie B. Rister J. Terrell D. Charlton-Perkins M. Pistillo D. (2013). Opposite feedbacks in the Hippo pathway for growth control and neural fate. *Science* 342:1238016. 10.1126/science.1238016 23989952 PMC3796000

[B19] Justice R. W. Zilian O. Woods D. F. Noll M. Bryant P. J. (1995). The Drosophila tumor suppressor gene warts encodes a homolog of human myotonic dystrophy kinase and is required for the control of cell shape and proliferation. *Genes. Dev.* 9 534–546. 10.1101/gad.9.5.534 7698644

[B20] Kango-Singh M. Nolo R. Tao C. Verstreken P. Hiesinger P. R. Bellen H. J. (2002). Shar-pei mediates cell proliferation arrest during imaginal disc growth in drosophila. *Development* 129 5719–5730. 10.1242/dev.00168 12421711

[B21] Kim J. Sitaraman S. Hierro A. Beach B. M. Odorizzi G. Hurley J. H. (2005). Structural basis for endosomal targeting by the Bro1 domain. *Dev. Cell* 8 937–947. 10.1016/j.devcel.2005.04.001 15935782 PMC2862258

[B22] Kok K. Naylor S. L. Buys C. H. (1997). Deletions of the short arm of chromosome 3 in solid tumors and the search for suppressor genes. *Adv. Cancer Res.* 71 27–92. 10.1016/s0065-230x(08)60096-2 9111863

[B23] Kwon Y. Vinayagam A. Sun X. Dephoure N. Gygi S. P. Hong P. (2013). The Hippo signaling pathway interactome. *Science* 342 737–740. 10.1126/science.1243971 24114784 PMC3951131

[B24] Lai Z. C. Wei X. Shimizu T. Ramos E. Rohrbaugh M. Nikolaidis N. (2005). Control of cell proliferation and apoptosis by mob as tumor suppressor, mats. *Cell* 120 675–685. 10.1016/j.cell.2004.12.036 15766530

[B25] Lu H. Bilder D. (2005). Endocytic control of epithelial polarity and proliferation in drosophila. *Nat. Cell Biol.* 7 1232–1239. 10.1038/ncb1324 16258546

[B26] Manteghi S. Gingras M. C. Kharitidi D. Galarneau L. Marques M. Yan M. (2016). Haploinsufficiency of the ESCRT component HD-PTP predisposes to cancer. *Cell Rep.* 15 1893–1900. 10.1016/j.celrep.2016.04.076 27210750

[B27] Matsudaira T. Mukai K. Noguchi T. Hasegawa J. Hatta T. Iemura S. I. (2017). Endosomal phosphatidylserine is critical for the YAP signalling pathway in proliferating cells. *Nat. Commun.* 8:1246. 10.1038/s41467-017-01255-3 29093443 PMC5665887

[B28] McCarter S. D. Johnson D. L. Kitt K. N. Donohue C. Adams A. Wilson J. M. (2010). Regulation of tight junction assembly and epithelial polarity by a resident protein of apical endosomes. *Traffic* 11 856–866. 10.1111/j.1600-0854.2010.01052.x 20214753 PMC3392093

[B29] McClatchey A. I. Fehon R. G. (2009). Merlin and the ERM proteins–regulators of receptor distribution and signaling at the cell cortex. *Trends Cell Biol.* 19 198–206. 10.1016/j.tcb.2009.02.006 19345106 PMC2796113

[B30] Miaczynska M. Pelkmans L. Zerial M. (2004). Not just a sink: endosomes in control of signal transduction. *Curr. Opin. Cell Biol.* 16 400–406. 10.1016/j.ceb.2004.06.005 15261672

[B31] Miura G. I. Roignant J. Y. Wassef M. Treisman J. E. (2008). Myopic acts in the endocytic pathway to enhance signaling by the Drosophila EGF receptor. *Development* 135 1913–1922. 10.1242/dev.017202 18434417 PMC2413058

[B32] Mu Z. Wang L. Deng W. Wang J. Wu G. (2017). Structural insight into the ragulator complex which anchors mTORC1 to the lysosomal membrane. *Cell Discov.* 3:17049. 10.1038/celldisc.2017.49 29285400 PMC5742854

[B33] Murphy J. E. Padilla B. E. Hasdemir B. Cottrell G. S. Bunnett N. W. (2009). Endosomes: a legitimate platform for the signaling train. *Proc. Natl. Acad. Sci. U.S.A.* 106 17615–17622. 10.1073/pnas.0906541106 19822761 PMC2764915

[B34] Odorizzi G. Katzmann D. J. Babst M. Audhya A. Emr S. D. (2003). Bro1 is an endosome-associated protein that functions in the MVB pathway in *Saccharomyces cerevisiae*. *J. Cell Sci.* 116 1893–1903. 10.1242/jcs.00395 12668726

[B35] Pan D. (2010). The Hippo signaling pathway in development and cancer. *Dev. Cell* 19 491–505. 10.1016/j.devcel.2010.09.011 20951342 PMC3124840

[B36] Pantalacci S. Tapon N. Leopold P. (2003). The salvador partner Hippo promotes apoptosis and cell-cycle exit in Drosophila. *Nat. Cell Biol.* 5 921–927. 10.1038/ncb1051 14502295

[B37] Robinson B. S. Huang J. Hong Y. Moberg K. H. (2010). Crumbs regulates salvador/warts/Hippo signaling in Drosophila via the FERM-domain protein expanded. *Curr. Biol.* 20 582–590. 10.1016/j.cub.2010.03.019 20362445 PMC2855393

[B38] Robinson B. S. Moberg K. H. (2011). Drosophila endocytic neoplastic tumor suppressor genes regulate sav/wts/hpo signaling and the c-Jun N-terminal kinase pathway. *Cell Cycle* 10 4110–4118. 10.4161/cc.10.23.18243 22101275 PMC3272291

[B39] Roux K. J. Kim D. I. Raida M. Burke B. (2012). A promiscuous biotin ligase fusion protein identifies proximal and interacting proteins in mammalian cells. *J. Cell Biol.* 196 801–810. 10.1083/jcb.201112098 22412018 PMC3308701

[B40] Saksena S. Sun J. Chu T. Emr S. D. (2007). ESCRTing proteins in the endocytic pathway. *Trends Biochem. Sci.* 32 561–573. 10.1016/j.tibs.2007.09.010 17988873

[B41] Salama N. R. Chuang J. S. Schekman R. W. (1997). Sec31 encodes an essential component of the COPII coat required for transport vesicle budding from the endoplasmic reticulum. *Mol. Biol. Cell* 8 205–217. 10.1091/mbc.8.2.205 9190202 PMC276074

[B42] Schmid E. M. McMahon H. T. (2007). Integrating molecular and network biology to decode endocytosis. *Nature* 448 883–888. 10.1038/nature06031 17713526

[B43] Shen X. Sun X. Sun B. Li T. Wu G. Li Y. (2018). ARRDC3 suppresses colorectal cancer progression through destabilizing the oncoprotein YAP. *FEBS Lett.* 592 599–609. 10.1002/1873-3468.12986 29364502

[B44] Szeles A. Yang Y. Sandlund A. M. Kholodnyuk I. Kiss H. Kost-Alimova M. (1997). Human/mouse microcell hybrid based elimination test reduces the putative tumor suppressor region at 3p21.3 to 1.6 cM. *Genes. Chromosomes Cancer* 20 329–336. 10.1002/(sici)1098-2264(199712)20:4<329::aid-gcc3>3.0.co;2-3 9408748

[B45] Taelman V. F. Dobrowolski R. Plouhinec J. L. Fuentealba L. C. Vorwald P. P. Gumper I. (2010). Wnt signaling requires sequestration of glycogen synthase kinase 3 inside multivesicular endosomes. *Cell* 143 1136–1148. 10.1016/j.cell.2010.11.034 21183076 PMC3022472

[B46] Tapon N. Harvey K. F. Bell D. W. Wahrer D. C. Schiripo T. A. Haber D. (2002). salvador Promotes both cell cycle exit and apoptosis in Drosophila and is mutated in human cancer cell lines. *Cell* 110 467–478. 10.1016/s0092-8674(02)00824-3 12202036

[B47] Toyooka S. Ouchida M. Jitsumori Y. Tsukuda K. Sakai A. Nakamura A. (2000). HD-PTP: a novel protein tyrosine phosphatase gene on human chromosome 3p21.3. *Biochem. Biophys. Res. Commun.* 278 671–678. 10.1006/bbrc.2000.3870 11095967

[B48] Trahair J. F. Wilson J. M. Neutra M. R. (1995). Identification of a marker antigen for the endocytic stage of intestinal development in rat, sheep, and human. *J. Pediatr. Gastroenterol. Nutr.* 21 277–287. 10.1097/00005176-199510000-00005 8523211

[B49] Udan R. S. Kango-Singh M. Nolo R. Tao C. Halder G. (2003). Hippo promotes proliferation arrest and apoptosis in the salvador/warts pathway. *Nat. Cell Biol.* 5 914–920. 10.1038/ncb1050 14502294

[B50] Wilson J. M. Whitney J. A. Neutra M. R. (1987). Identification of an endosomal antigen specific to absorptive cells of suckling rat ileum. *J. Cell Biol.* 105 691–703. 10.1083/jcb.105.2.691 3305521 PMC2114780

[B51] Xu J. Vanderzalm P. J. Ludwig M. Su T. Tokamov S. A. Fehon R. G. (2018). Yorkie functions at the cell cortex to promote myosin activation in a non-transcriptional manner. *Dev. Cell* 46:e5. 10.1016/j.devcel.2018.06.017 PMC6086586 30032991

[B52] Xu T. Wang W. Zhang S. Stewart R. A. Yu W. (1995). Identifying tumor suppressors in genetic mosaics: the Drosophila lats gene encodes a putative protein kinase. *Development* 121 1053–1063. 7743921 10.1242/dev.121.4.1053

[B53] Yan Y. Denef N. Tang C. Schupbach T. (2011). Drosophila PI4KIIIalpha is required in follicle cells for oocyte polarization and Hippo signaling. *Development* 138 1697–1703. 10.1242/dev.059279 21429988 PMC3074446

[B54] Yu F. X. Guan K. L. (2013). The Hippo pathway: regulators and regulations. *Genes. Dev.* 27 355–371. 10.1101/gad.210773.112 23431053 PMC3589553

[B55] Yue T. Tian A. Jiang J. (2012). The cell adhesion molecule echinoid functions as a tumor suppressor and upstream regulator of the Hippo signaling pathway. *Dev. Cell* 22 255–267. 10.1016/j.devcel.2011.12.011 22280890 PMC3288783

[B56] Zhao B. Li L. Lei Q. Guan K. L. (2010). The Hippo-YAP pathway in organ size control and tumorigenesis: an updated version. *Genes. Dev.* 24 862–874. 10.1101/gad.1909210 20439427 PMC2861185

